# Peroxiredoxins in Cancer and Response to Radiation Therapies

**DOI:** 10.3390/antiox8010011

**Published:** 2019-01-01

**Authors:** Tom E. Forshaw, Reetta Holmila, Kimberly J. Nelson, Joshua E. Lewis, Melissa L. Kemp, Allen W. Tsang, Leslie B. Poole, W. Todd Lowther, Cristina M. Furdui

**Affiliations:** 1 Department of Internal Medicine, Section on Molecular Medicine, Wake Forest School of Medicine, Winston-Salem, NC 27157, USA; tforshaw@wakehealth.edu (T.E.F.); rholmila@wakehealth.edu (R.H.); atsang@wakehealth.edu (A.W.T.); 2 Department of Biochemistry, Wake Forest School of Medicine, Winston-Salem, NC 27157, USA; kinelson@wakehealth.edu (K.J.N.); lbpoole@wakehealth.edu (L.B.P.); tlowther@wakehealth.edu (W.T.L.); 3 The Wallace H. Coulter Department of Biomedical Engineering, Georgia Institute of Technology and Emory University, Atlanta, GA 30332, USA; joshlewis@gatech.edu (J.E.L.); melissa.kemp@bme.gatech.edu (M.L.K.)

**Keywords:** peroxiredoxin, radiation resistance, ionizing radiation, oxidative stress, transcriptomics, proteomics, TCGA, NCI-60

## Abstract

Peroxiredoxins have a long-established cellular function as regulators of redox metabolism by catalyzing the reduction of peroxides (e.g., H_2_O_2_, lipid peroxides) with high catalytic efficiency. This activity is also critical to the initiation and relay of both phosphorylation and redox signaling in a broad range of pathophysiological contexts. Under normal physiological conditions, peroxiredoxins protect normal cells from oxidative damage that could promote oncogenesis (e.g., environmental stressors). In cancer, higher expression level of peroxiredoxins has been associated with both tumor growth and resistance to radiation therapies. However, this relationship between the expression of peroxiredoxins and the response to radiation is not evident from an analysis of data in The Cancer Genome Atlas (TCGA) or NCI60 panel of cancer cell lines. The focus of this review is to summarize the current experimental knowledge implicating this class of proteins in cancer, and to provide a perspective on the value of targeting peroxiredoxins in the management of cancer. Potential biases in the analysis of the TCGA data with respect to radiation resistance are also highlighted.

## 1. Introduction

Reactive oxygen species (ROS), and in particular hydrogen peroxide (H_2_O_2_), are essential regulators of cellular signaling, metabolism and epigenetics. The main endogenous sources of ROS are the mitochondria and NADPH oxidases (NOX; NADPH: nicotinamide adenine dinucleotide phosphate, reduced state), both generating superoxide that is then converted to H_2_O_2_ and oxygen by subcellularly localized superoxide dismutases [1,2,3]. The regulation of redox state is fundamental for cell survival and various antioxidant systems regulate redox metabolism and the levels of H_2_O_2_ under physiological conditions [3,4,5]. Environmental factors, such as exposure to toxins, heavy metals, radiation, pathogens, and others (e.g., diet), can overwhelm the antioxidant capacity of the cell and result in high intracellular ROS and oxidative stress [6,7]. Excessive cellular ROS have been well defined as damaging to cellular components such as DNA, proteins and lipids and as important factors linking environmental exposures to diseases ranging from cardiovascular and neurological diseases to diabetes, aging and cancer [6,8], the focus of this review. 

As a consequence of altered cellular metabolism, cancer cells are generally characterized by increased glycolysis and higher levels of ROS [4]. Tumor growth and the often associated inflammation can further increase ROS and shift the redox balance towards a more oxidative state [6]. At low to moderate levels, ROS may contribute to carcinogenesis either by acting as signaling molecules, inducing DNA mutations and genomic instability, or inactivating tumor suppressor genes. At high levels, ROS promote cellular damage and death, a principle exploited in cancer treatment with ionizing radiation (IR) and chemotherapies (e.g., anthracyclines) [4]. 

The increased ROS levels in the cancerous cells often lead to an adaptive increase in the expression of antioxidant defense proteins (e.g., peroxiredoxins) allowing the cells to survive and grow under conditions of increased metabolism [4]. Tumors resistant to radiation therapies are also typically characterized by higher expression of antioxidant proteins but in this case associated with *lower* levels of ROS. Thus, antioxidant systems, including peroxiredoxins, have emerged as new therapeutic targets in the fight against cancer. We will review herein the structural and mechanistic aspects of peroxiredoxins relevant for their function in the regulation of cancer redox metabolism, with a particular focus on the implications for radiation therapies.

## 2. Peroxiredoxins as Cellular Antioxidant and Signaling Proteins

Peroxiredoxins, abbreviated as either Prx or Prdx, are ubiquitous, highly expressed antioxidant enzymes forming up to 1% of cellular protein content [9]. Prxs catalyze the reduction of H_2_O_2_, peroxynitrite (ONOO^−^), and various organic peroxides (ROOH) to water, nitrite or hydroxyl derivatives (ROH), respectively, with high catalytic efficiency (rate constants ~10^7^ M^−1^s^−1^). Prxs are the dominant proteins reacting with H_2_O_2_, and 10,000 times more H_2_O_2_ is estimated to react with Prxs than with glutathione [10]. Due to the high kinetic efficiency of reaction with H_2_O_2_, mammalian Prxs not only protect against oxidative damage, but also play significant roles in regulating the cellular redox environment and modulating signal transduction and metabolic pathways. A number of studies have linked high Prxs levels with the cancer phenotype [11], with radiation resistance [12,13,14], and with poor prognosis for chemotherapy [15]. 

### 2.1. Subcellular Distribution of Prx Isoforms and Catalytic Cycle

Human cells contain six Prx isoforms that are found in different subcellular compartments and have been assigned to three classes based on structural and sequence characteristics (Table 1) [16,17,18]. 

**Figure 1:**
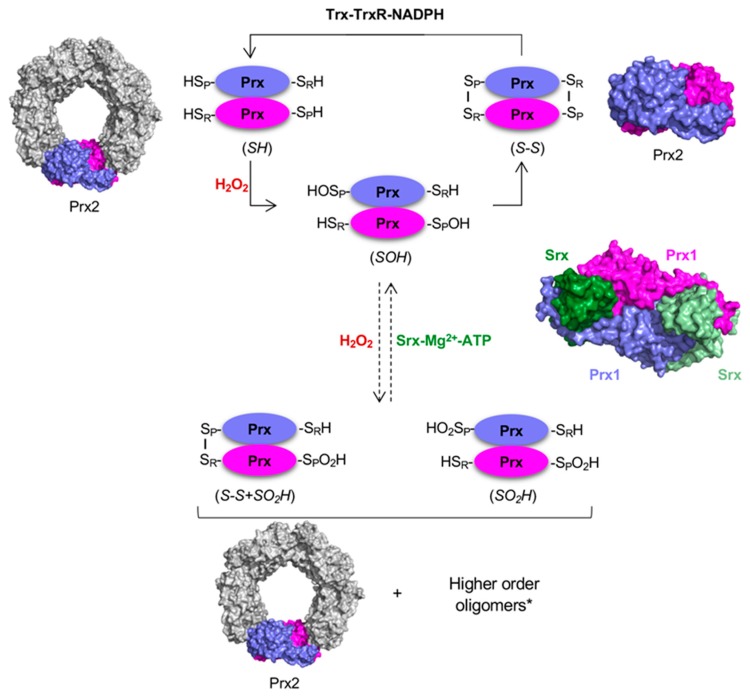
Catalytic cycle of Prx1 class with typical 2-Cys mechanism. The peroxidatic cysteine (C_P_, -S_P_H) is oxidized by H_2_O_2_ to form cysteine sulfenic acid (-S_P_OH). During normal catalysis, this intermediate forms a disulfide with the resolving cysteine (C_R_, -S_R_H). In Prx1-4, the C_R_ is located near the C-terminus and forms an intermolecular disulfide bond with its partner subunit in the obligate homodimer. Reduction of this disulfide is performed by the thioredoxin-thioredoxin reductase-NAPDH (Trx-TrxR-NADPH) system. As H_2_O_2_ concentrations increase, one or both peroxidatic cysteine residues may be hyperoxidized and inactivated as the -S_P_OH intermediate reacts with a second or third molecule of H_2_O_2_ to form cysteine sulfinic acid (-S_P_O_2_H) or cysteine sulfonic acid (-S_P_O_3_H), respectively. Sulfiredoxin (Srx) is able to repair hyperoxidized Prxs in the presence of Mg^2+^ and ATP. PDB codes: Prx2, 1QMV [19]; Prx1-Srx complex, 2RII [20]).

During the first step of catalysis for all Prxs (Figure 1), the highly conserved peroxidatic cysteine (C_P_) reacts with a substrate peroxide and is oxidized to a short lived sulfenic acid intermediate (-S_P_OH/-S_P_O^−^). This intermediate is resolved by the reaction with either a cysteine (C_R_) on a different Prx monomer (typical 2-Cys), on the same monomer (atypical 2-Cys), or a reducing small molecule such as glutathione (1-Cys), to produce inter- or intramolecular disulfides (C_P_-C_R_, C_P_-G), and in the process releasing water. In the case of human Prx1–5, the disulfide can be rapidly reduced back to the -SH by the thioredoxin (Trx) and Trx reductase (TrxR) system using NADPH as electron donor. 

In humans, the three classes (Prx1, Prx5, and Prx6) are associated with three mechanisms of disulfide resolution based on the presence and location of the C_R_: typical 2-cys (Prx1–4), atypical 2-cys (Prx5), and 1-cys Prxs (Prx6) (Table 1) [18,21]. While this designation has been helpful as a way to describe individual Prxs, it is problematic to use these terms as a substitute for the Prx structural classes when considering Prxs across biology genera. When present, the C_R_ has so far been found in five different locations in the protein structure with four of them falling under the atypical 2-Cys designation [22]. Prx1 proteins almost exclusively use a typical 2-Cys mechanism, but there is significant diversity in the location of the C_R_ within the other Prx classes. The Prx5 and Prx6 classes have both 1-Cys and 2-Cys members and < 20% of Prx5 proteins have the C_R_ in the same location as human Prx5 [17]. 

Critically, under high H_2_O_2_ conditions, a second H_2_O_2_ molecule can react with the -S_P_OH (-S_P_O^−^) to induce further oxidation (termed hyperoxidation) to the sulfinic acid (-S_P_O_2_H/-S_P_O_2_^−^), leading to inactivation of the peroxidase activity of Prxs. The sensitivity to hyperoxidation is isoform dependent and has been studied most comprehensively for Prx1 class members. Mitochondrial Prx3 has been shown to be more resistant to hyperoxidation relative to the Prx1 and Prx2 isoforms, which may be critical for the function of Prx3 in the mitochondria [23,24]. Hyperoxidized Prxs can be reduced by sulfiredoxin in an ATP-dependent reaction to allow re-entry into the catalytic cycle (Figure 1) [20,25]. Thus, the level, timing and subcellular localization of hyperoxidized Prxs are also regulated in cells. These observations have led to the discovery of new functions for Prxs (e.g., phosphorylation signaling, unfolded protein repair) in addition to the established role as regulators of H_2_O_2_ levels. 

### 2.2. Prx Oligomeric State

It is important to note that in solution, the Prx enzymes are found in various oligomeric states depending on the specific isoform and redox state (Table 2) [18]. These oligomeric states are critical for the function of Prxs as chaperones or for redox interactions with other proteins. Both Prx5 and Prx6 have been shown to form homodimers in solution [18]. Prx5 is dimeric regardless of the oxidation state [26], while there is some data to suggest that oxidation of Prx6 leads to destabilization of the dimer and formation of monomeric species [27]. Typical 2-Cys Prxs (Prx1–4) exist in an equilibrium between dimer and decamer (5 dimers) or dodecamer (6 dimers) in solution (Table 2). The fully folded (FF), reduced active state favors decamer/dodecamer formation. Upon reaction with peroxides, the regions containing the C_P_ and C_R_ become locally unfolded (LU) to allow for intermolecular disulfide formation between the two subunits in the obligate homodimer. This rearrangement destabilizes the decamer/dodecameric state and shifts the equilibrium toward a dimeric state [18,21]. The oligomeric state of typical 2-Cys Prxs is also influenced by other conditions including pH (increasing from pH 7 to pH 8 destabilizes decamers/dodecamers), ionic strength, presence of His-tag, and protein concentration (higher concentrations favor decamer/dodecamer formation) [28,29].

## 3. Peroxiredoxins in H_2_O_2_ Sensing, Signaling and Protein Folding

**Figure 2:**
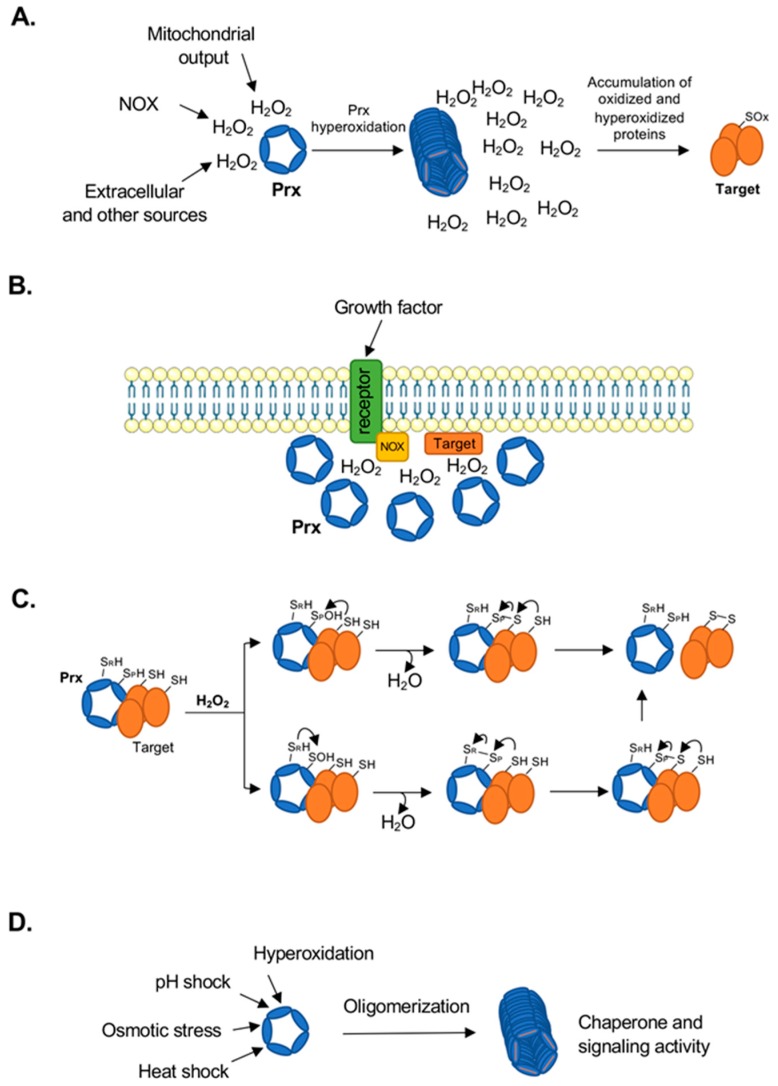
Cellular functions of peroxiredoxins. (**A**) The floodgate hypothesis proposes that hyperoxidation of Prx enzymes decreases localized peroxidase activity, which may allow for an accumulation of H_2_O_2_ for oxidation of less sensitive signaling proteins (SOx). (**B**) Prx may act as a gatekeeper to maintain H_2_O_2_ concentrations within a precise intracellular location for specific signaling requirements. (**C**) Oxidized Prxs may directly relay oxidative signals to other proteins. (**D**) A variety of cellular signals can cause Prx to stack into multiple decamer/dodecamer rings in tube-like formations which possess chaperone activity.

The role of Prx enzymes is not constrained solely to their peroxidase activity, but also expands to encompass other related functions such as sensors of H_2_O_2_ levels, regulators of signaling and metabolism, oxidation of redox sensitive signaling proteins, and protein folding. There are several lines of evidence supporting these functions, which will be described here (Figure 2) [37,38].

### 3.1. Sensors of H_2_O_2_ Levels

The kinetics of each step in the catalytic cycle of Prxs is fine-tuned to match the function and subcellular location of each isoform. For example, Prx2 is most sensitive to hyperoxidation-dependent inactivation followed by Prx1 and much later the mitochondrial Prx3 matching the higher generation of ROS in the mitochondria and the need to protect this key organelle from oxidative damage [39,40]. In the cytosol, three peroxiredoxins (Prx1, Prx2, and Prx5) coexist and at low H_2_O_2_ Prx1 and Prx2 react more rapidly with H_2_O_2_ than Prx5. However, as H_2_O_2_ concentrations rise, the amount of reduced Prxs decreases and eventually the peroxidase activity becomes limited by either disulfide formation or reduction. Based on kinetic analysis, this shift in the rate limiting step occurs first for Prx2, followed by Prx1, and lastly by Prx5 [40,41]. The oxidized Prxs (-SOH state or disulfide-linked dimers) can then either serve as redox sensors or initiate staggered redox relays by interacting with redox sensitive proteins.

### 3.2. Regulators of Signaling and Metabolism

The ‘floodgate’ model proposes that while basal H_2_O_2_ concentrations are rapidly reduced by Prx to maintain a reducing environment, rapid and localized increases in H_2_O_2_ may cause hyperoxidation of Prx and subsequent loss of peroxidase activity [38]. This further increases H_2_O_2_ levels locally allowing for redox modification and regulation of less sensitive proteins within this redox microenvironment (Figure 2A). Another mechanism by which localized Prx inhibition can occur is through phosphorylation of either Tyr194 or Thr90 (Prx1 numbering), modifications which have been shown to occur in cells through Src-family kinases or cyclin-dependent kinases, respectively [42,43]. In such cases of localized Prx inhibition (by either hyperoxidation or phosphorylation), the localized accumulation of H_2_O_2_ which can no longer react with nearby Prxs can instead modify redox-sensitive signaling proteins (e.g., protein tyrosine phosphatases) that are recruited to the vicinity of signaling complexes like the receptor/NOX complex, promoting signaling through additional phosphorylation events [22] (Figure 2B). Loss of Prx activity through phosphorylation has been shown to promote signaling through growth factors like PDGF and EGF [43], and at the centrosome during mitosis [44]. The role of hyperoxidation as a signaling-relevant modification is less clear, but several studies have provided evidence that hyperoxidation can occur as a result of biological signaling events. For example, it has been shown that the H_2_O_2_ produced by CYP11B1 during corticosterone synthesis inactivates Prx3 through hyperoxidation, and the resulting accumulation of H_2_O_2_ initiates a negative feedback loop that is sustained until repair of the Prx3 by mitochondrially-associated sulfiredoxin occurs, processes that display a circadian oscillation [45]. It has also been proposed that Prxs act as a buffer to contain mitogenic signaling H_2_O_2_ within the local cellular environment and maintain specificity [46]. This model of Prx hyperoxidation as a signaling-relevant modification has been challenged by computational studies predicting that hyperoxidation of Prxs and the resulting saturation of the local environment with H_2_O_2_ is too slow to enable regulation of signaling proteins which are comparatively poorly reactive towards H_2_O_2_ [47], although one caveat of this computational approach is the lack of clarity regarding how high localized H_2_O_2_ concentrations can reach during cell signaling processes.

### 3.3. Propagators of Redox Signaling

An alternative proposed signaling role of Prx enzymes is in the initiation and transmission of redox events through the transfer of disulfides between proteins, also known as ‘redox relays’ (Figure 2C) [48]. This has been recently reviewed in detail [48] and is only briefly described here. In this pathway, the redox sensitive Prx proteins are first oxidized by low levels of H_2_O_2_. This oxidation is then transferred to a second less sensitive signaling protein through the formation of a mixed disulfide bond between the interacting protein and the Prx; this mixed disulfide could either occur by direct reaction with the -S_P_OH or by disulfide exchange with the C_P_-C_R_ bond. This has been evidenced both in vitro [49,50,51] and predicted computationally [47]. However, this model also suggests that removal of Prxs would result in decreased protein oxidation (disulfides), rather than the expected increase resulting from higher cellular H_2_O_2_ concentrations. This has proven difficult to demonstrate experimentally due to instability of many oxidative protein modifications and potential compensation by other antioxidant proteins. This model also suggests that sulfenylation would not be observed on signaling proteins since only the Prx protein would be expected to form -SOH which is contradicted by proteomic approaches utilizing specific sulfenylation probes that show formation of these oxidized species on multiple cellular proteins [52,53]. 

### 3.4. Protein Folding/Chaperone Activity

Aside from the functions described above, Prxs have also been assigned protein chaperone activity (Figure 2D). Specifically, hyperoxidation or posttranslational modifications of human Prxs induce the formation of high molecular weight (HMW) stacked multimeric rings which do not have peroxidase activity but gain ATP-independent chaperone or holdase activity [35,54] (Table 2 and Table 3, and references therein). Several examples of this have been observed for hyperoxidized human and yeast Prxs [35,54,55], while mutagenesis studies suggest phosphorylation of Prx1 at Thr90 promotes the formation of HMW species and induces chaperone activity [56]. In contrast, glutathionylation at Cys83 (a non-catalytic Cys found at the dimer-dimer interface) has been shown to disrupt decamer formation and to inhibit the Prx1 chaperone activity [57]. An exception to this appears to be Prx3 for which structural analysis under different pH conditions suggests that the protein forms self-chaperoning structures, which retain peroxidase activity under low pH [58]. Although in some cases the loss of peroxidase activity at high H_2_O_2_ concentrations may appear counterproductive, experiments in yeast found that the hyperoxidized, HMW Prxs are no longer utilizing Trx, freeing Trx for other cell survival functions [59]. An interesting addition to the chaperone function was more recently described for membrane associated Prx2 in erythrocytes [60]. A small fraction of Prx2, independent of its redox state, was shown to bind to the cell membrane and to inhibit the aggregation of partially unfolded and oxidized hemoglobin. Although much remains to be understood about the mechanism of this interaction, the presence of Prx2 was shown to protect against hemolytic anemia resulting from hemoglobin oxidation and Heinz body formation, but the mechanism of this protection would differ from the HMW chaperone activity [60].

## 4. Peroxiredoxins in Cancer

**Figure 3:**
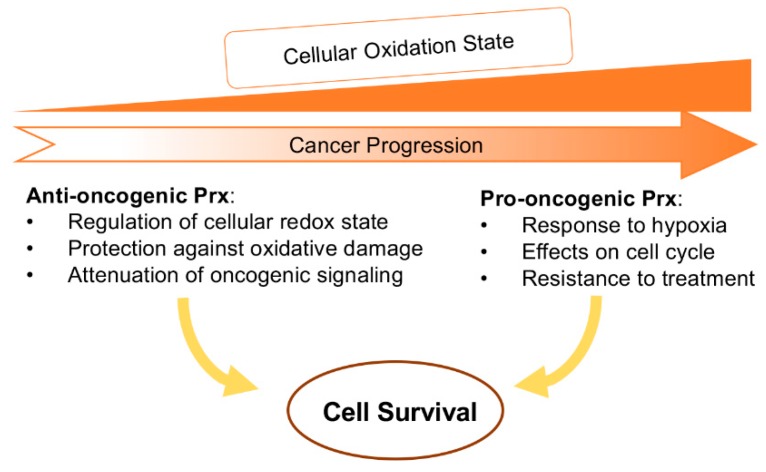
Role of peroxiredoxins in carcinogenesis. Prxs can act in either anti- or pro-oncogenic manner depending on the cellular context.

As discussed above, increased production of ROS including H_2_O_2_ can act as mitogenic [63,64] or genotoxic [65,66] signals. Thus, antioxidants may either promote or protect against pro-oncogenic pathways depending on the cancer stage and redox metabolic state. The loss of the Prx antioxidant function in the individual gene knockouts of Prx1 [67,68] and Prx5 [69] has been shown to induce tumorigenesis. In contrast, the addition of antioxidants like N-acetylcysteine and vitamin E increased lung cancer progression [70] and melanoma metastasis [71]. Consistent with these latter findings, in advanced tumors there may be a more pronounced oxidative shift caused by increased metabolic activity required to support proliferation. Factors such as hypoxia and inflammation would also contribute to this state. In these more advanced tumors, the role of Prxs may switch to being pro-tumorigenic protecting cancer cells from death. This shift in the redox state with tumor progression has been reviewed in depth (e.g., [72]) and it will be reviewed here only in the context of Prxs as both anti-carcinogenic and pro-carcinogenic double-edged sword (Figure 3).

### 4.1. Prx Expression in Cancer and Potential for Targeted Therapeutics

A survey of studies analyzing Prx expression in human cancer reveals that Prxs are upregulated in many human cancers at both mRNA and protein levels, although it should be noted that in a small number of cases it was also downregulated (see Hampton et al. [73]). This was also observed in vitro using cells in culture and animal models. For example, in a cell line series of breast cancer progression, Prx6 expression was increased in the most metastatic cell line, while Prx6 stable knockdown xenografts exhibited decreased tumor growth and metastasis [74]. Furthermore, in a comparison between normal liver cells and several liver cancer cell lines, Prx3 mRNA and protein expression was significantly increased [75]. In general, it may be best to consider Prxs as predominantly pro-cell survival – survival of healthy tissue or diseased. Importantly, even in cells that upregulate Prxs, the ROS levels are higher than the normal cells, and these cancerous cells do not have the same capacity to respond to ROS like a normal cell [4]. Therefore, inhibition of Prx activity is expected to lead to a further increase in ROS and cell death for cancer cells but not normal surrounding cells, which have multiple compensatory mechanisms of response to increased ROS. Thus, there should be a significant therapeutic index for Prx inhibitors in the treatment of cancer. In support of this concept, the novel compound AMRI-59 is a selective inhibitor of Prx1 and is significantly more cytotoxic to a transformed ovarian cancer cell line compared with the parental non-tumorigenic cell line [76]. Prx3 can be irreversibly inhibited by the natural product thiostrepton, which is again significantly more cytotoxic in vitro to malignant mesothelioma cells over both primary and immortalized mesothelial cells [77,78].

### 4.2. Peroxiredoxins as Protectors Against DNA Damage

Both in vitro and in vivo studies have demonstrated a role for Prxs in the prevention of oxidative DNA damage and neoplastic transformation. Prx1 [67,68] and Prx3 [79] knockout mice exhibit increased DNA oxidation (specifically, 8-oxo-2’-deoxyguanosine) without addition of a ROS-inducing or DNA damaging agent [80]. This modification could lead to increased genomic instability and oncogenesis [81]. Furthermore, both human Prx1 [82,83] and the *S. cerevisiae* Prx homologue Tsa1 [84] bind to telomeric DNA and regulate telomerase activity [82,83,84]. While Prx2 was also observed in enriched human telomere chromatin, its regulatory effect in this context has not been characterized [82]. Interestingly, the impact of the respective Prxs on telomere structure differs significantly between the two species. In humans, Prx1 associates with telomere chromatin and decreases H_2_O_2_ oxidation of DNA at guanine (8-oxo-2’-deoxyguanosine), a modification known to prematurely terminate telomere extension and cause cytotoxic effects [82,83]. Blocking this interaction is a particularly attractive target for cancer therapy given the increased levels of ROS [4] and the high demand for DNA replication in cancer cells [83]. Conversely, *S. cerevisiae* deficient in Tsa1 show no clear DNA lesions and have irregular telomere lengthening [84]. It has been hypothesized that this difference between species is due to an increased ability of *S. cerevisiae* to withstand oxidative stress [83]. In addition to this influence on telomeres, Prx2 has been proposed to act as a redox sensor and slow DNA replication in response to oxidative metabolic signals [85]. Specifically, Prx2 was shown to interact with the TIMELESS protein which is a component of the replication protection complex. An increase in ROS, including H_2_O_2_, was shown to be associated with a slowdown in DNA synthesis at the replication fork but not necessarily lower dNTP concentrations. Increased ROS led to a decrease in chromatin bound Prx2, as well as dissociation of TIMELESS from the replisome, leading to the hypothesis that Prx2 serves as a redox sensor regulating DNA replication [85]. This complex may again be an attractive target for cancer therapy as cancer cells preferentially replicate DNA at a slower rate to retain genome integrity [85]. It is intriguing to consider how the redox regulation of SAMHD1 [86] and other enzymes [87] in dNTP metabolism may be involved in regulation of DNA replication and whether this may also involve interactions with Prxs. 

Furthermore, the Prx5 isoform was found to decrease damage of both mitochondrial and nuclear DNA following treatment with either H_2_O_2_, tert-butylhydroperoxide (tBHP), or by metal catalyzed H_2_O_2_ generation [88,89]. Radiation also generates multiple ROS and products of lipid peroxidation, and thus Prxs may protect cells from DNA damage caused by exposure to radiation (environmental or therapeutic). The role of Prxs in the response to cancer radiation therapy will be discussed later in this review.

### 4.3. Peroxiredoxins in Oncogenic Signaling

As previously described, peroxiredoxins have cell signaling functions beyond their enzymatic peroxidase function. Transgenic mice have been an excellent tool to demonstrate this. Following the introduction of HRAS, KRAS, and HPV8 oncogenes, mice with knockout of either Prx1 [68,90,91] or Prx6 [92] show increased tumor growth, suggesting that these Prxs can attenuate oncogenic signaling. Multiple non-exclusive mechanisms have been elucidated and proposed for the Prx-dependent suppression of oncogene-induced carcinogenesis. Several studies described here found that Prxs are closely involved in growth factor signaling in a redox dependent manner through various pathways. This again highlights the intrinsic importance of Prxs in oncogenic cell signaling and makes this class of proteins an attractive target for cancer therapy.

The first Prx1 knockout mouse model found increased protein tyrosine phosphorylation in tumors [67], a common pro-oncogenic signature [93]. Cellular oxidation is a well-known activator of protein tyrosine kinases (PTK, though a select number of kinases are also inhibited by oxidation) [94] and inhibitor of protein tyrosine phosphatases (PTP) [94,95], and a number of experiments have found a direct role for Prx in tyrosine phosphorylation signaling. For example, the pro-proliferation oncoprotein c-Abl is activated by oxidative signals and, although the exact mechanism has not yet been elucidated, there is evidence of Prx1 binding to c-Abl and inhibiting its activation [96,97,98] in a process reversible by H_2_O_2_ [99]. 

Oxidation of Prx1 was found to change selectivity of its binding to different protein tyrosine phosphatases [100]. Reduced Prx1 was found to bind both p38MAPKα kinase phosphatase (MKP) 1 and 5, protecting these from the deactivating effects of H_2_O_2_. However, oxidation of Prx1 decreased binding to MKP1 and increased binding to MKP5, resulting in exclusive deactivation of MKP5. This change in MKP activation ratio was hypothesized to inhibit apoptosis and increase the cancer phenotype through p38MAPKα signaling, a known driver of oncogenesis [100,101].

Prx1 was also observed to interact with the well-known oncogene c-myc. An early study found that Prx1 interacted with the regulatory domain of c-Myc in rat fibroblasts, significantly changing the expression profile of c-Myc targets and decreasing the ability to form tumor-like colonies [102]. A follow-up investigation with Prx1 knockout mice again found that c-Myc activity was dysregulated, although mRNA and protein expression levels of the transcription factor were unaffected. Thus, dysregulation was considered to be a cancer risk factor which required further oncogenic processes to cause neoplastic transformation [68]. Similarly, c-Myc activity and oncogenicity in prostate cancer can be attenuated by Akt phosphorylation in a redox dependent manner, but unlike the earlier studies this was linked to increased c-Myc proteolysis rather than transcriptional dysregulation [103]. Although Prxs were not investigated in this study, H_2_O_2_-induced Akt phosphorylation is well established [104,105,106] and phosphorylation of Akt is increased by overexpression of Prx1 [107], Prx2 [108], and Prx6 [109,110]. Added to these effects, H_2_O_2_ was also determined to selectively inhibit the kinase activity of Akt2, but not of other Akt isoforms [106]. These findings are significant because each of Prx1-4 [111,112,113,114] is upregulated in prostate cancer, and Prx6 is associated with increased reoccurrence of prostate cancer [115]. These data further substantiate the function of Prx1 and other peroxiredoxins in the Akt-c-Myc oncogenic pathway.

### 4.4. Peroxiredoxins and Hypoxia in Cancer

Hypoxia is a significant decrease in cellular O_2_ concentration (<2%) compared to the physiological normoxic concentrations (≈5%, depending on tissues). As a tumor becomes more advanced and larger, the oxygen demand can surpass the oxygen supply which has important implications for proliferation, metabolism, senescence, metastasis, and response to chemoradiation therapies [116]. Hypoxia is primarily detected and responded to by hypoxia inducible factors (HIFs), with H_2_O_2_ being required for HIF1α stabilization [117]. Although this mechanism of HIF1α activation during hypoxia is debated [118,119,120], experiments using isolated mitochondria, electron transport chain inhibitors, and catalase overexpression support the notion that hypoxia stimulates a release of ROS signals from the mitochondria which cause downstream stabilization of HIF1α [119,120,121]. This increase in cellular ROS may also lead to an adaptive increase in the expression of Prxs. Several lines of evidence support this concept. 

Using the A549 lung cancer cell line it was found that the Prx1 gene, a target of the antioxidant transcription factor Nrf2 [122], was upregulated under hypoxic conditions along with Trx [123]. Additionally, in a mouse xenograft model of oral squamous cell carcinoma, larger tumors were found to have hypoxic centers with increased Prx1 expression [124,125]. Similarly, in human hepatocarcinoma and pancreatic cancer, Prx1 expression was positively associated with tumor size and microvascular invasion [126,127], which may also be suggestive of the role of Prx1 in cancer progression. Interestingly, while overexpression of Prx3 protected thymoma cells in vitro from hypoxia mediated apoptosis [128], Prx3 was downregulated by HIF1α during hypoxia in another cell line model [129]. 

Neovascularization is stimulated by hypoxia through increased vascular endothelial growth factor (VEGF) expression [130]. Knockdown of Prx1 decreased total VEGF levels and vascularization of prostate cancer in a mouse subcutaneous xenograft model [111]. Through mutagenesis studies, this was found to be mediated by an interaction with toll-like receptor 4, in a manner independent of the Prx1 peroxidase activity [111]. In addition, VEGF pathway can also be regulated by Prx2 at the VEGF receptor (VEGFR) in human vascular cells [131]. Activation of VEGFR increased cellular ROS in both control and Prx2 knockdown cells; however, knockdown of Prx2 decreased autophosphorylation and thus activation of VEGFR. Furthermore, this process was dependent on Prx2 peroxidase activity and association with the membrane indicating again a role of Prx2 in modulating tyrosine phosphorylation signaling described above. Taken together these studies demonstrate the important role of Prxs in the vascularization response to hypoxia, which has relevance in both cancer progression and response to therapies.

Hypoxia impacts metabolism as the cell adapts to the decreased oxygen concentrations [132]. In particular, HIF-mediated expression of glucose transporters and pyruvate dehydrogenase kinases is increased in hypoxic cells. These changes promote the Warburg effect [132] through increased glucose uptake and decreased mitochondrial respiration [133,134,135,136]. However, HIF-regulated transcription of SLC2A3 (glucose transporter 3) and PDK3 (pyruvate dehydrogenase lipoamide kinase isozyme 3) genes can be inhibited by Prx2 and Prx4 interaction with HIF, independent of their peroxidase activity [137]. Interestingly, Prx2 expression was also increased under hypoxia, suggesting that Prx2 may negatively regulate the shift to Warburg metabolism under sustained hypoxia [137]. It is surprising to note that although mitochondrial redox metabolism is important in both hypoxia signaling and TCA cycle activity [119,120,121,138], very little is known about Prx3 regulation of these pathways. However, overexpression of Prx3 in mice improves glucose tolerance, increasing Akt and GSK3 phosphorylation which has implications for glucose transport [139]. In complementary studies, Prx3 knockout mice showed decreased glucose tolerance and decreased expression of mitochondrial biogenesis proteins. Furthermore, expression of Prx5 modified to be solely targeted to the mitochondrial matrix decreased the output of hypoxia-induced H_2_O_2_ from the mitochondria to the cytosol [120]. Expression of this Prx5 construct also decreased the influx of Ca^2+^ into the mitochondria during acute hypoxia, but further metabolic implications have not been explored [120].

Hypoxia is also important for acquired cancer resistance to radiation therapies [140], a topic discussed in more detailed below. Early studies found that oxygen concentration was important for cell sensitivity to radiation, with well-oxygenated tumors being more responsive to radiation therapy [141,142,143]. This was considered to be due to the irreversible and irreparable reaction of DNA lesions with molecular oxygen, termed the oxygen fixation hypothesis [144]. However, more recent studies suggest that this may not be the sole mechanism of resistance to radiation injury [145]. Indeed, as indicated above, hypoxia increases glucose uptake and glycolysis and increased flux through the pentose phosphate pathway is important for resistance to radiation [146,147]. The increased production of NADPH through this pathway may contribute to resistance by maintaining levels of active, reduced Prx through the Trx/TrxR and glutathione reductase systems [148,149], while also supplying the building blocks for the repair of damaged DNA.

## 5. Peroxiredoxins in Radiation Treatment of Cancer

### 5.1. Ionizing Radiation

Ionizing radiation (IR) is widely used to treat many types of cancer and acts by inducing DNA damage, ROS, and other reactive species (e.g., e^−^_aq_, •OH, H•, H_2_O_2_) [150,151]. With the exception of H_2_O_2_, the radical species are unstable and in the presence of oxygen, e^−^_aq_ and H• radicals are rapidly converted to superoxide/perhydroxyl (O_2_•^−^/HO_2_•) species [152,153]. In biological systems, organic radicals (R•) are also generated, leading to formation of hydroperoxides (ROOH) such as in lipid peroxidation [152]. Besides increasing ROS levels, IR can also stimulate the activity of nitric oxide synthase (NOS), increasing nitric oxide (•NO) and peroxynitrite (ONOO^−^) levels [152]. 

The initial burst of ROS with ionizing radiation was found to persist several hours after exposure [4,150,153]. This sustained increase in ROS may be partly due to the irradiation-induced accumulation of cells in the G2/M phase which have the highest mitochondrial content and activity of the cell cycle phases [153]. These mitochondria may also be damaged by IR leading to further leakage of electrons from the mitochondrial electron transfer chain resulting in excess ROS generation [152]. Furthermore, NOX, another important source of ROS, is also activated by radiation exposure, leading overall to persistent oxidative stress and contributing to cell death [4]. 

### 5.2. Mechanisms of Resistance to IR

There are several key factors that modify the cellular response and determine overall efficacy of radiation treatment in cancer. These include efficiency of DNA repair, cell cycle distribution, tumor repopulation, tumor oxygenation (as cancer tissues are more hypoxic than normal tissues) [154,155], and other intrinsic tumor features including the expression levels of ROS-scavenging antioxidant systems [4]. In particular, cells are most sensitive to irradiation during G2/M phase, less sensitive during G1 and S phase, and least sensitive during late S phase, which has been hypothesized to be at least partly due to increased activity of homologous repair mechanisms particularly during late S phase cycle [156,157,158]. 

Hormesis can broadly be defined as an adaptive response to an otherwise toxic agent, by which a chronic low dose of such an agent has a beneficial effect to health [159]. Low doses of radiation might also be beneficial leading to enhanced patient survival and lower cancer burden through mechanisms of hormesis, as shown in some studies [160]. This could be due to the stimulation of the immune system as well as adaptive response to oxidative stress [161]. However, there are contradicting studies where low-dose radiation showed either no effect or an increase in cancer incidence, and other studies indicating that low-dose radiation does not enhance, or even inhibits, repair of DNA double-strand breaks [160,161]. To our knowledge, the role of Prxs in radiation hormesis has not been studied, but *C. elegans* Prx2 was implicated in the hormetic response to metformin, a ROS inducing drug [162]. 

### 5.3. Peroxiredoxins and Response to Cancer Radiation Therapy

As ROS are key effectors of IR treatment and the Prxs are main enzymes in regulation of cellular H_2_O_2_, there has been increasing interest in studying the role of Prxs in the response to radiation therapies. For patients with head and neck cancer (HNC), it was found that tumors with increased expression of Prx2 are significantly more resistant to radiation therapy compared to matched tumors with a lower expression of Prx2, and that IR induced expression of Prx2 in HNC cell lines [163]. Another group found that both Prx2 and Prx4 were upregulated with IR treatment in two HNC cell lines [164]. Furthermore, in a matched model of radiation resistance for HNC, our group also found upregulation of several peroxiredoxins (Prx1, Prx2, Prx3 and Prx6) and decreased ROS species in radiation resistant cells compared to matched radiation sensitive cells [14]. These expression patterns were also found in other in vivo and in vitro models with IR treatment. Irradiation of mouse intestine in vivo increased expression of Prx1 protein [165]. In colon cancer and glioma cells in vitro Prx1 protein and mRNA was increased with IR treatment in a dose and time dependent manner [166]. This response does not seem to be restricted to cancerous tissue, as IR treatment of mouse testis increased expression of both Prx1 and Prx2 [167]. However, the expression of Prx1, Prx2, and Prx4 was not increased following up to 16 Gy of radiation treatment in myeloid leukemia cells, although there was increased oxidation of Prx1 and Prx2 observed by monitoring dimer/monomer ratios on a non-reducing SDS-PAGE gel [168]. The lack of changes in Prx expression was rationalized as being due to the already high basal expression of these proteins and the radiation resistance phenotype of these cells.

The mechanistic implications of increased expression of Prxs in radiation resistant cancer cells and tumors have also been explored using molecular approaches. Park et al. [169] found that expression of Prx4 was higher in a radiation resistant HNC cell line compared with a separate radiation sensitive HNC cell line. Knockdown of Prx4 in the radiation resistant line and overexpression of Prx4 in the sensitive cell line reversed the response to radiation, sensitizing the resistant cell line to radiation and increasing the radiation resistance of the sensitive cell line [169]. This was also found for Prx2 in matched breast cancer and glioma cell lines [12,170]. Similarly, knockdown or overexpression of Prx1 decreased or increased cell viability following radiation treatment, respectively, in colon cancer and glioma cell lines [166]. 

The impact of knockdown alone of Prx enzymes was studied extensively in radiation resistant cancer cell lines. Knockdown of Prx1 decreased cell growth and antioxidant capacity of a colon cancer cell line, while sensitizing cells to IR treatment [171]. In two lung cancer cell lines, knockdown of Prx1 both in vitro and in vivo xenografts not only decreased cell growth and metastasis, but also increased radiation efficacy (measured as post-irradiation growth delay), independently of p53 expression status [172]. In a different study, overexpression of Prx1 alone was able to decrease apoptosis in irradiated lung cancer cells through suppression of the JNK pathway [173]. Interestingly, the same effect was noted with the peroxidase-inactive Cys52Ser mutant, highlighting, as discussed above, that Prxs have important cellular functions beyond peroxidase activity. In a mouse model, overexpression of Prx6 ameliorated skin damage following irradiation [174]. However, irradiation increased the levels of microRNA miR-214 which binds to Prx6 mRNA and decreases translation of the protein [174]. This observation suggests that following a traumatic IR insult Prx6 may be involved in the decision-making process between cell survival and cell death.

Another Prx-mediated mechanism of radiation resistance could be related to Prx regulation of cell cycle. As previously stated, cells are more sensitive to IR in the G2/M phase, which is also the phase with the highest mitochondrial content. In both human and rat glioma cells, knockdown of Prx2 increased cell doubling time and significantly affected cell cycle progression [12]. Specifically, in the rat glioma cells there were fewer Prx2 knockdown cells in S phase and more in G2/M phase, while in the human glioma cells there were fewer Prx2 knockdown cells in G1 phase and more in G2 phase along with increased phosphorylation of Cdc2 [12]. On the other hand, there was no difference in cell cycle progression between matched radiation resistant and sensitive HNC cells, although 2 Gy IR induced G2/M arrest in the radiosensitive cell line which had lower expression of Prx1 and Prx2 [14].

Taken together these studies indicate that Prxs are important modifiers of cancer progression and radiation response and could potentially be an attractive target to improve the efficacy of radiation therapy. Nevertheless, although inhibitors of Prxs have been developed, there are currently no reports of their application as radiation sensitizers.

### 5.4. Prx Expression and Radiation Resistance in Cancer Databases

**Figure 4:**
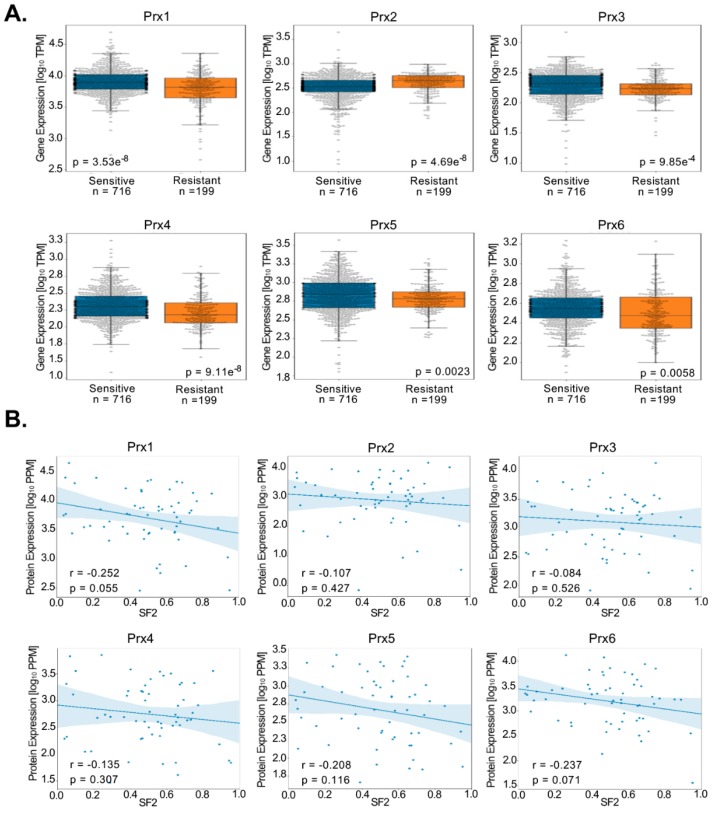
TCGA and NCI-60 analysis of Prx expression. (**A**) Analysis of TCGA data shows expression of Prx2, but no other Prxs as significantly increased in radiation resistant tumors. Sample size and significance are individually listed for each dataset. (**B**) Analysis of NCI-60 data show no statistically significant association of any Prxs with the radiation response using SF2 as an indicator of radiation response.

Given the multitude of experimental studies linking expression of Prxs with response to radiation treatment, we sought to investigate this association using information in The Cancer Genome Atlas (TCGA; RNA-Seq) and NCI-60 panel of cancer cell lines (iBAQ proteomics) [175,176]. In the analysis of TCGA data, we assigned the radiation sensitive phenotype to patients who had complete or partial response to radiation treatment (based on the RECIST criteria) and the radiation resistant phenotype to patients with stable disease or radiographic progressive disease [177]. RNA-Seq data from TCGA samples normalized as transcripts per million (TPM) was taken directly from the Gene Expression Omnibus (accession number GSE62944) [175]. Surprisingly, the results using data across tumor types show that only Prx2 is significantly upregulated (p = 4.69 × 10^−8^) in radiation resistant tumors, and in fact the other Prxs are significantly downregulated (Figure 4A). This contrasts sharply with the aforementioned experimental data in cell culture and animal studies which established the function of Prxs in cancer. There are clearly experimental aspects in the collection of both clinical and in vitro data that could be the cause of these differences in the expression pattern of Prxs. The difference in oxygen levels between physiologic pO_2_ in vivo conditions and 20% O_2_ cell culture conditions could impact redox metabolism to a greater extent than other metabolic or signaling pathways, leading overall to a more redox-responsive state. Additionally, given the high level of tumor heterogeneity for many cancers, cell lines isolated from radiation sensitive or resistant tumors may not necessarily represent the bulk tumor and its overall radiation response phenotype. The cells growing in vitro could simply be the cells that adapt the best to the specific cell culture conditions. With the TCGA analysis, there are also potential questions regarding the contribution of other cell types present in the tumor (e.g., immune cells, fibroblasts) to the gene expression signature, the time point of tissue collection for gene expression analysis relative to the time of radiation treatment and RECIST categorization, the availability of only gene expression data given the knowledge that differences in protein levels are not always reflected in gene expression, and the very low number of radiation resistant tumors in the database. These issues increase the difficulty to perform statistical analysis of radiation sensitive versus resistant tumors (e.g., in the case of HNC, 124 radiation sensitive and only 15 radiation resistant tumors) in TCGA.

To better understand the discrepancy between TCGA data and data from cells in culture or animal xenografts, we next conducted an analysis of the iBAQ protein data available for the NCI-60 panel of cancer cell lines [176]. Non-normalized peptide-level iBAQ data from NCI-60 samples were taken from the EMBL-EBI database (project number PXD005940) [176]. After performing regularized expectation maximization (RegEM) imputation, iBAQ data were normalized to parts per million (PPM), and peptides corresponding to a single NCBI gene symbol were summed into a single protein expression value [178]. There was no significant association between the protein level of any Prxs with the radiation response of these cell lines using surviving fraction at 2 Gy (SF2) as an indicator of radiation sensitivity [179]. 

Both the TCGA and NCI-60 analyses integrated samples from multiple tissue types (Figure 4) and Prx expression levels may vary to a greater extent based on cancer type than based on radiation sensitivity. Specifically, the large proportion of lower grade glioma samples within the radiation resistant group (111/199, 55.8%) compared to the radiation sensitive group (40/716, 5.6%) in the TCGA analysis may confound any changes in Prx expression based on radiation sensitivity. However, the limited number of radiation sensitive and resistant samples within individual cancer types in the TCGA and NCI-60 databases restricts the ability for identifying statistically significant differences in expression between these two phenotypes. Improved collection and categorization of tumor samples exposed to IR and assessed for radiation response would greatly enhance the capability to study the role of Prx expression in radiation resistance.

## 6. Conclusions

Prx proteins play a central role in maintaining cellular redox balance in part by regulating H_2_O_2_ levels. Persistent imbalances in the cellular redox state can lead to diseases, such as cancer, which often show higher oxidative state compared to normal cells. Cancerous cells commonly adapt to higher ROS by increasing the expression of Prxs and other antioxidant systems. Accordingly, Prxs are upregulated in many cancers, where they participate in virtually all processes from tumor growth and metastasis to regulating the response to cancer therapies. This is different from physiological/pre-oncogenesis conditions, where Prxs seem to have a more protective role in decreasing DNA damage and oncogenic signaling. Therefore, the function of Prxs changes from anti-oncogenic to pro-oncogenic during cancer progression and this has clinical implications. Indeed, as advanced cancers are characterized by higher ROS, it has been logically proposed that antioxidant treatment would slow progression [180,181]. However, many clinical trials using antioxidants have been ineffective or negative [70,71,182,183,184,185] indicating that the mechanisms of development are far more complex than anticipated. Instead, perturbation of cellular antioxidant systems may be a better option to selectively target cancer cells, which are under increased oxidative stress, while sparing healthy cells which have other compensatory mechanisms [77,78,186]. Selective targeting of Prxs may also sensitize cancer cells to radiation therapy and should be investigated further.

## Figures and Tables

**Table 1:** The six mammalian peroxiredoxin (Prx) proteins classified based on structural and mechanistic properties.

Prx Protein	Prx1	Prx2	Prx3	Prx4	Prx5	Prx6
**Class**	Prx1				Prx5	Prx6
**Mechanism**	Typical 2-Cys				Atypical 2-Cys	1-Cys
**Localization**	Cytosol Nucleus	Cytosol Lipid membranes Nucleus	Mitochondria	ER/Golgi Extracellular	Cytosol Mitochondria Peroxisome	Cytosol

**Table 2:** Oligomeric state of the Prx enzymes in solution in various oxidation states.

Prx Class	Prx Protein	Oxidation State	Favored Oligomeric State in Solution	Size on Non-Reducing SDS-PAGE	References
Prx1	Prx1, 2, & 4	SH (reduced)	Decamer	Monomer	[18,29]
	Prx3	SH (reduced)	Dodecamer	Monomer	[30]
	Prx1, 2, 3, & 4	SS (oxidized)	Dimer	Dimer	[18,29]
	Prx1, 2, 3, & 4	SOH (oxidized)	n.d.	Monomer ^1^	
	Prx1, 2, 3, & 4	SO_2_H (hyperoxidized)	Decamer & high order oligomers ^2^	Can run as either dimer (1 SO_2_H, 1SS in dimer) or monomer (2 SO_2_H per dimer)	[19]
Prx5	Prx5	SH (reduced)	Dimer	Monomer	[18]
	Prx5	SS (oxidized)	Dimer	Monomer	[18]
Prx6	Prx6	SH (reduced)	Dimer	Monomer	[31,32]
	Prx6	SOH, SO_2_H (oxidized)	Dimer and monomer	Monomer	[32,33,34]

^1^ In the absence of an alkylating agent, -S_P_OH will react rapidly with any available thiol under non-reducing, denaturing conditions leading to non-native disulfides. ^2^ Higher order oligomers of Prx1 and 2 are associated with the gain of a chaperone-like function [35,36]. SDS-PAGE: sodium dodecyl sulfate polyacrylamide gel electrophoresis.

**Table 3:** Effects of selected post-translational modifications on the oligomeric state of Prx1 subfamily members.

Protein	Modification	Effect on Oligomeric State	References
Prx1	Glutathionylation-C83 (Non-catalytic Cys at dimer/dimer interface)	Destabilizes decamer	[57]
	p-Thr90	Favors decamer and higher order oligomers	[56]
	p-Tyr194	No change in SS oligomers	[43]
	C_P_-S-nitrosylation	Destabilizes decamer	[61]
Prx2	Tyr nitration by ONOO^−^	Destabilizes decamer	[62]
